# Long Non-Coding RNA NEAT1 Associates with SRp40 to Temporally Regulate PPARγ2 Splicing during Adipogenesis in 3T3-L1 Cells

**DOI:** 10.3390/genes5041050

**Published:** 2014-11-27

**Authors:** Denise R. Cooper, Gay Carter, Pengfei Li, Rehka Patel, James E. Watson, Niketa A. Patel

**Affiliations:** 1 Research Service, J. A. Haley Veterans Hospital, 13000 Bruce B. Downs Blvd, Tampa, FL 33612, USA; E-Mails: gay.carter@va.gov (G.C.); jwatson@health.usf.edu (J.E.W.); npatel@health.usf.edu (N.A.P.); 2 Department of Molecular Medicine, Morsani College of Medicine, University of South Florida, Tampa, FL 33612, USA; E-Mails: pli@health.usf.edu (P.L.); rpatel15@health.usf.edu (R.P.)

**Keywords:** lncRNA, NEAT1, 3T3-L1 cells, adipogenesis, SR proteins

## Abstract

Long non-coding (lnc) RNAs serve a multitude of functions in cells. NEAT1 RNA is a highly abundant 4 kb lncRNA in nuclei, and coincides with paraspeckles, nuclear domains that control sequestration of paraspeckle proteins. We examined NEAT1 RNA levels and its function in 3T3-L1 cells during differentiation to adipocytes. Levels of NEAT1 transcript, measured by RT-PCR, fluctuated in a temporal manner over the course of differentiation that suggested its role in alternative splicing of PPARγ mRNA, the major transcription factor driving adipogenesis. When cells were induced to differentiate by a media cocktail of insulin, dexamethasone, and isobutylmethyxanthine (IBMX) on Day 0, NEAT1 levels dropped on Day 4, when the PPARγ2 variant was spliced and when terminal differentiation occurs The appearance of PPARγ2 coordinates with the PPARγ1 variant to drive differentiation of adipocytes. SiRNA used to deplete NEAT1 resulted in the inability of cells to phosphorylate the serine/arginine-rich splicing protein, SRp40. SiRNA treatment for SRp40 resulted in dysregulation of PPARγ1 and, primarily, PPARγ2 mRNA levels. SRp40 associated with NEAT1, as shown by RNA-IP on days 0 and 8, but decreased on day 4, and concentrations increased over that of IgG control. Overexpression of SRp40 increased PPARγ2, but not γ1. Although lncRNA MALAT1 has been investigated in SR protein function, NEAT1 has not been shown to bind SR proteins for phosphorylation such that alternative splicing results. The ability of cells to increase phosphorylated SR proteins for PPARγ2 splicing suggests that fluxes in NEAT1 levels during adipogenesis regulate alternative splicing events.

## 1. Introduction

Alternative splicing of pre-mRNA during adipogenesis is a means of ushering preadipocytes towards fully differentiated adipocytes by expanding the diversity of the proteome that accompanies maturation of the cells. There are substantial numbers of pre-mRNA that are alternatively spliced before and during adipogenesis, and the potential function of long non-coding RNAs during differentiation has not been incorporated into this complex scenario of gene expression. Peroxisome proliferator activated receptor γ (PPARγ) is an important driver of adipogenesis. The two splice variants—PPARγ1 and PPARγ2—are generated by alternate first exon usage.

More than half the mammalian transcriptome consists of noncoding (nc) RNA. These regulatory RNAs can be classified into small (18–200 nt) and long non-coding (lnc) RNAs (200 nt to >100 kb) [1]. They are cDNAs without any positive-strand open reading frames longer than 30 amino acids [2]. LncRNAs regulate gene expression by diverse mechanisms that are not yet fully understood [3] and can confer a degree of spatial specificity that is not possible to achieve with small RNAs such as microRNAs or proteins [4]. While only a few functions of lncRNAs have been revealed to date, they control every level of gene expression. By regulating post-transcriptional gene regulation they control protein synthesis, RNA maturation, transport, and gene silencing via regulating chromatin structure. The ability of lncRNAs to recruit RNA binding proteins, one of the largest protein classes, to gene promoters expands the regulatory repertoire available to the transcriptional program [5]. The prominent protein factors that bind to scaffolding lncRNAs are SR (serine/arginine rich) proteins. SR proteins have a modular structure with one or two N-terminal RNA recognition motifs (RRMs) and an arginine/serine repeat (RS) domain [6]. RNA splicing events are controlled by changes in the concentration and localization of SR proteins by signaling pathways [7]. One such mechanism of regulating their localization is their phosphorylation by the phosphoinositide-3 kinase/Akt/Clk kinase pathway [8].

LncRNAs can act as components of nuclear bodies by providing a scaffolding to organize nuclear structures. In this role, they regulate gene expression by controlling pre-mRNA processing and export [9]. The lncRNA, NEAT1 (also known as MEN epsilon/β or nuclear-enriched abundant transcript), plays an essential role in the assembly and architecture of paraspeckles in mouse and human cells [10,11,12]. MALAT1, a.k.a. NEAT2, lies immediately downstream of NEAT1 on chromosome 11 [13], but there is independent regulation of expression of these lncRNAs [14]. NEAT1 and MALAT1 (metastasis-associated lung adenocarcinoma transcript 1) appear to serve as structural docking sites for accumulating specific splicing factors such as SR proteins [15]. This class of lncRNA is retained within the nucleus and localizes to specific nuclear bodies. Nuclear bodies do not represent major sites of transcription or splicing, but are thought to be sites of assembly, modification, and storage of pre-mRNA splicing machinery [16]. Speckles are sites that house SR factors that are then “recruited” to sites of active transcription [17]. MALAT1 was found to be a gene that was up-regulated in metastatic non-small-cell lung cancer cells [18]. MALAT1 regulated alternative splicing by modulating SR protein phosphorylation [19]. NEAT1 was identified in a screen where XIST and MALAT1 were also identified [13]. Early studies showed that NEAT1 and MALAT1 function in messenger RNA metabolism. NEAT1, however, localized to the periphery of nuclear speckles and associated with the SR protein, SC35 splicing domain [13]. NEAT1 is a large lncRNA with a transcript of approximately 4 kb in length [13]. A functional role for it was shown through its appearance in paraspeckles, which are adjacent to nuclear speckles containing MALAT1 in cultured cell lines [10].

Prior studies focused on the location and role of NEAT1 in nuclear bodies as scaffolds [4,9,20]. Here, we further hypothesize that NEAT1 associates with the serine-arginine rich “SR” protein, SRp40, so that Clk kinase phosphorylates it for regulating alternative splicing of pre-mRNA during adipocyte differentiation. After its phosphorylation, SRp40 is “released” from the lncRNA, possibly by decaying or exporting the lncRNA. Hence, an increase in the concentration of free, phosphorylated SRp40 occurs near the spliceosome. To demonstrate this involvement of NEAT1, we used siRNA to deplete NEAT1, and examined splice variants for PPARγ2 and γ1, the main drivers of adipogenesis. For this functional attribute, we identified SRp40 as the SR protein that regulates splicing of PPARγ2 and γ1. We show varying levels of the lncRNA during the temporal differentiation of adipocytes that reflect a decrease of lncRNA on Day 4 of adipogenesis. The knockdown of NEAT1 with siRNA attenuated phosphorylation of SRp40 by Clk1 kinase. This study increases our knowledge of how varying levels of NEAT1 can function in the timing of alternative splicing of primary transcripts regulating adipogenesis.

## 2. Experimental

### 2.1. Materials

Purified antibodies to SR proteins were raised to our specifications and recognized only one band on Western blots (WB). They have been characterized previously [21], and are available from EMD Millipore, Billenca, MA, USA. MAb104 (ATCC) recognizes the phosphoepitope of all SR proteins that are Clk substrate motifs [21]. PPARγ and β-actin antibodies were from Cell Signaling (Boston, MA, USA).

### 2.2. Cell Culture

3T3-L1 murine pre-adipocytes (from ATCC) were grown to confluence and on Day 0 were cultured in DMEM (high glucose with 10% newborn calf serum at 37 °C and 10% CO_2_, with insulin, dexamethasone, and isobutyl-1-methylxanthine. On Day 2, media was replaced with DMEM HG, 10% FBS, and bovine insulin. On Day 4, cells were cultured in DMEM HG plus 10% FBS. Culture media was then changed every 2 days.

### 2.3. Immunoprecipitation and Western Blotting

Immunoprecipitation and Western blotting of SR proteins was performed as described [21].

### 2.4. siRNA/Antisense Knockdown

SiRNA to NEAT1 and scrambled siRNA were purchased from Ambion (Grand Island, NY, USA). Small interfering RNA were transfected into cells using the Lonza nucleofector (Lonza Group Ltd., Basel, Switzerland) apparatus. Knockdown of RNA is verified by rtPCR for NEAT1.

### 2.5. RNA Isolation and Quantitative Real-Time RT-PCR

Total RNA was isolated using RNA-Bee (Tel Test Center, Friendswood, TX, USA) as recommended by the manufacturer. Total RNA was reverse transcribed using random hexamers (Life Technologies, Grand Island, NY, USA) and Omniscript reverse transcription kit (Qiagen, Valencia, CA, USA). The primers for NEAT1 sense were TTGGGACAGTFFACGTGTGG (5' to 3') and antisense was TCAAGTCCAGCAGAGCA (5' to 3') [22]. GAPDH was used for standardization with sense TGACGTGCCGCCTGGAGAAC, and antisense CCGGCATCGAAGGTGGAAGAG [23]. For PPARγ1, the sense primer was GAGTGTGACGACAAGATTG (5' to 3') and the PPARγ2 sense primer was TCTGGGAGATTCTCCTGTTGA (5' to 3'). The antisense primer was common, GGTGGGCCAGAATGGCATTCT (5' to 3') [24]. After optimization of primer concentrations, 2.0 μL cDNA was amplified using Maxima SYBR Green/ROX qPCR master mix (Thermo Scientific, Pittsburgh, PA, USA) and an ABI 7900 HT thermal cycler (Applied Biosystems, Life Technologies, Grand Island, NY, USA) using standard settings. Relative RNA (RQ) levels were generated by SDS v 2.0 software [25] (ABI) using the ΔΔ Ct method with GAPDH as the endogenous control and Day 0 as the calibrator sample.

### 2.6. RNA-IP Assay (RIPA)

The RIP kit was purchased from Sigma (Sigma Aldrich, St. Louis, MO, USA) and the protocol was followed as per manufacturer’s instructions after cellular differentiation for 0, 4 and 8 days. The SRp40 antibody was from our lab, SNRNP70 antibody from Millipore (EMD Millipore, Merck KGaA, Darmstadt, Germany), and the IgG antibody was included in the kit. Ten percent cell lysate was removed for input sample. Immunoprecipitation was performed with 2 micrograms SRp40 antibody, SNRNP70 antibody (positive control) or IgG antibody (as negative control). RNA was purified and treated with DNAse to remove genomic DNA. SYBR Green Real-time qPCR was performed as described above using NEAT1 primers and primers for U1 RNA, the binding partner for the positive control SNRNP70. The yield (% input) and specificity (fold enrichment) was calculated using Excel™ template for RIP from Sigma.

## 3. Results and Discussion

### 3.1. Expression of PPARγ during 3T3-L1 Adipogenesis

**Figure 1:**
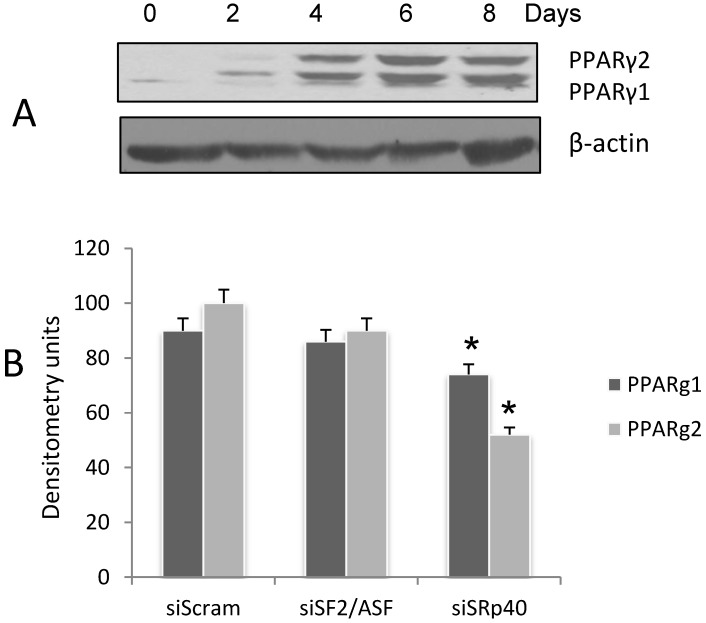
The splicing of PPARγ1 and γ2 in differentiating 3T3-L1 adipocytes is decreased with siRNA to SRp40. (**A**) Cell lysates were collected on days as indicated, and subjected to WB using anti-PPARγ. β-actin was probed on the same blot as a control. The experiment was repeated three times with similar results; (**B**) Cells were treated with siRNA as indicated. SiSF2/ASF and siSRp40 produced a reduction of >70% in the target proteins (data not shown [29]). Cell lysates were collected on Day 4 for Western blot analysis of the PPARγ splice variants. Densitometry scans of the Western blot are plotted as arbitrary units as a result of siRNA treatment. The experiment was repeated three times with similar results. Data were analyzed with two-way ANOVA (Prism6 software [30]) and siSRp40 significantly (*p > 0.0001) reduced both PPARγ1 and γ2 as indicated with an asterisk.

PPARγ is the primary gene product driving adipogenesis in progenitor cells. Two PPARγ splice variants, PPARγ1 and PPARγ2 have been cloned from mouse and human [26,27]. The forms differ only in the NH2-terminal with PPARγ2 expressing an additional 30 amino acids. When cells are placed in a cocktail of insulin, dexamethasone and IBMX to initiate differentiation, PPARγ1 is the first splice variant to be detected. On day 4, when cells are undergoing terminal differentiation, PPARγ2 is detected (Figure 1A). The two variants are detected on WB. Exon B is spliced into the final transcript, and PPARγ2 is expressed. If exon B is skipped, then the alternative splicing results in a shorter isoform, PPARγ1. The cassette exons A1-A2 are not translated. The highest expression level of both variants is in fat [28].PPARγ2 appears as the upper band on the blot, reflecting the translation of exon B, and PPARγ1 appears as the lower band.

### 3.2. SRp40 Functions in the Splicing of PPARγ1 and γ2

The SR proteins involved in the splicing of PPARγ1 and PPARγ2 have not previously been identified. There are specific binding motifs for SR proteins in each of the three exons involved in alternative splicing. Since adipocyte differentiation is initiated by addition of a cocktail containing insulin, we evaluated select SR proteins based on their potential roles (or lack thereof), in insulin induced alternative splicing. To elucidate the SR proteins involved in PPARγ exon selection, ASF/SF2 (SFRS1), and SRp40 (SFRS5) proteins were targeted for depletion using siRNA in 3T3L1 pre-adipocytes. Cells were harvested on Day 4, when both splice variants are detected, and the levels of PPARγ1 and γ2 were quantified as a function of siRNA depletion. SRp40 depletion (>60% by RT-PCR analysis, data not shown [29]) decreased the expression of both splice variants, but more so for PPARγ2 (Figure 1B). ASF/SF2 depletion had no effect. We focused further on SRp40 since a role for it has been shown in insulin mediated alternative splicing during adipogenesis [8].

**Figure 2:**
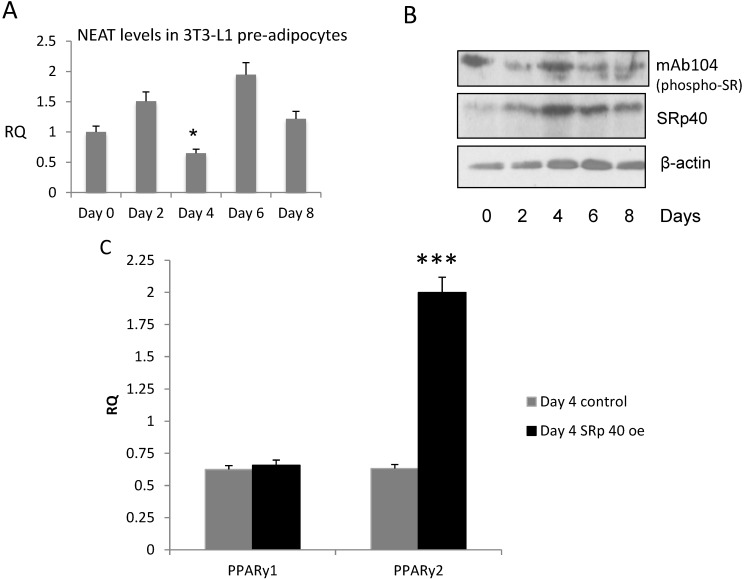
Regulation of NEAT1 and SRp40 proteins during adipogenesis. A. Levels of NEAT1 in 3T3-L1 cells during differentiation as described in Figure 1A. (**A**) Cellular RNA was extracted as described here (Experimental Section 2.5), and levels of NEAT1 and MALAT1 were quantified by qRT-PCR. The asterisk denotes a significant decrease in NEAT1 on day 4 (*p* > 0.0001) when compared to day 2 and 6; (**B**) Cell lysates were also collected and probed for SRp40 protein by Western blot during 12 days of adipogenesis; (**C**) SRp40 plasmid (2 µg) was transfected into 3T3-L1 cells on day -1, prior to induction of adipogenesis. Total RNA was isolated and subjected to qRT-PCR. Day 0 Control (Ctrl) was the calibrator sample. The experiments were repeated four times with similar results. Two-way ANOVA analysis indicated that PPARγ2 was increased (***p > 0.0001) as a result of SRp40 overexpression on day 4.

### 3.3. NEAT1 and SRp40 Levels during Adipogenesis

NEAT 1 lncRNA in differentiating 3T3-L1 cells was reported in a GEO dataset (GSE6794) by the Friedman lab [31]. We confirmed its occurrence in the 3T3-L1 cells, and found that NEAT1 levels were lower on Day 4 compared to Days 0, 2 or 6 (Figure 2A). SRp40 concentrations are maximal on Day 4 of adipogenesis (Figure 2B). These blots represent both phosphorylated (with anti-mAb104) and total protein (anti SRp40). Phosphorylated SRp40, detected using mAb104, depicts an increase in phospho-SRp40 on Day 4 (upper panel). To evaluate the role of SRp40 in PPARγ splicing during adipogenesis, SRp40 (2 μg) was overexpressed in 3T3L1 pre-adipocytes. On Day 4, using real-time qPCR, our results indicated PPARγ2 expression was >2-fold higher than in control cells (Figure 2C).

### 3.4. NEAT1 Down-Regulation Decreased SRp40 Phosphorylation

**Figure 3:**
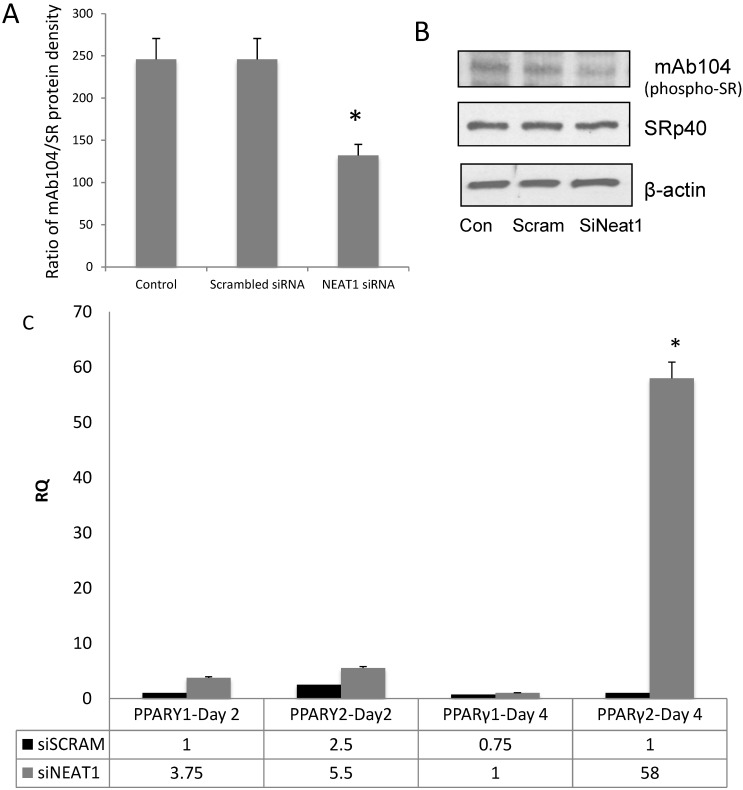
Depletion of NEAT1 decreases phosphorylation of SRp40. 3T3L1 cells were transfected with NEAT1 siRNA (25 nM) and whole cell lysates were collected. (**A**) The ratio of phosphorylated SRp40* vs.* SRp40 concentrations are shown after treatment of 3T3-L1 cells with scrambled siRNA or NEAT1 siRNA. Data was analyzed using ANOVA with *p* > 0.0001 for siNEAT1 treatment; (**B**) Western blots of phosphorylated SRp40, total SRp40 and β-actin on day 4 of adipogenesis; (**C**) 3T3L1 cells were transfected with NEAT1 siRNA or scrambled siRNA and analyzed with qRT-PCR for PPARγ1 and PPARγ2 levels. The experiments were repeated three times with similar results. Day 4 PPARγ2 levels were significantly (* *p* < 0.0001) higher than day 0 (ANOVA analysis).

Although another lncRNA, MALAT1, has been shown to be involved in sequestering SR proteins, the scaffolding of SRp40 by NEAT1 has not been shown. We previously showed that Clk1 (cdc2-like kinase 1) phosphorylated SRp40 in 3T3L1 cells during adipogenesis. To determine the role of NEAT1 in SRp40-mediated PPARγ alternative splicing, 3T3-L1 cells were treated with siRNA for NEAT1. Whole cell lysates were prepared on Day 4 to determine if phosphorylation of SRp40 by Clk, was diminished. This was demonstrated using mAb104, a monoclonal antibody known to recognize Clk kinase motifs in SR proteins [21]. When compared to the blot of total SRp40 protein, phosphorylation of SRp40 was attenuated (Figure 3A,B). In separate experiments, NEAT1 was deleted with siRNA and levels of PPARγ1 and γ2 were measured by qRT-PCR. Results show increased PPARγ2 levels were increased more than 60-fold with NEAT1 knockdown (Figure 3C).

### 3.5. NEAT1 Associated with SRp40

**Figure 4:**
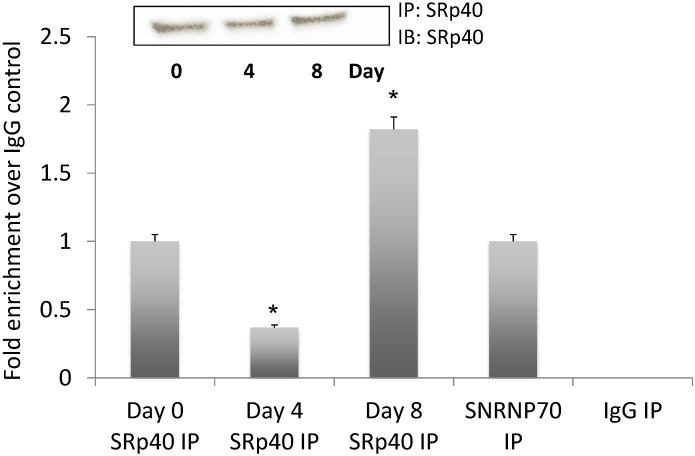
NEAT1 associated with SRp40 protein on days 0, 4, and 8 of adipogenesis. Cell lysates were immunoprecipitated with SRp40 antibody using the RIP assay kit from Sigma. SNRNP70, positive, and negative IgG controls were run on days 0, 4, and 8. Quantitative RT-PCR analysis of NEAT1 is expressed as fold enrichment over the IgG control. The blot of SRp40 IP is shown as an inset. This experiment was repeated three times with similar results.

If NEAT1 serves to “tether” SRp40, we reasoned that it should continue to do so during adipogenesis. For this experiment, we selected Days 0, 4, and 8. Following RNA-IP, Figure 4 demonstrates the detection of NEAT1 RNA associated with SRp40. The decrease in SRp40 interacting with NEAT1 on Day 4 is anticipated as this is when high concentrations of the phosphorylated protein are recruited to the exons for splice site selection. The enrichment on Day 8 is also anticipated as NEAT1 continues to modulate the amount of free phosphorylated SRp40.

### 3.6. SRp40 Concentrations and Phosphorylation Increased on Day 4 of Adipogenesis

**Figure 5:**
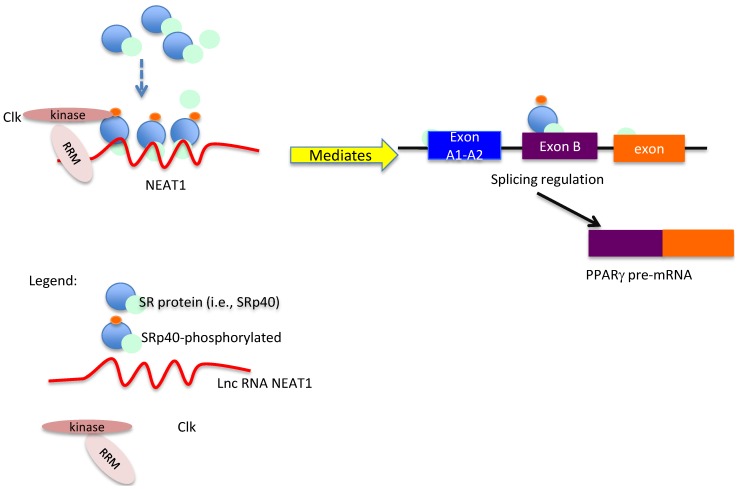
NEAT1 associates with SRp40 to potentiate its phosphorylation by Clk kinase (presumably in paranuclear bodies). This schematic shows how NEAT1 can tether SR proteins and, specifically, SRp40, and Clk kinase prior to its release and role in PPARγ splicing regulation.

Alternative splicing is regulated by the concentration and phosphorylation of splicing factors. When cell lysates were monitored for SR protein levels during adipogenesis, we detected an increase in SRp40 concentrations by western blot during the progression of adipogenesis in 3T3-L1. We hypothesized that the release of phosphorylated SRp40 during this crucial time in adipogenesis should result in nuclear reorganization with the splicing of PPARγ2. Our previous study demonstrated phosphorylation of SRp40 by Clk kinase in 3T3-L1 cells. Taken together, we diagrammed (Figure 5) the potential scheme of how NEAT1 scaffolding and association with SR proteins could result in titrating their nuclear concentration and phosphorylation for splicing.

## 4. Conclusions

The broad distribution of NEAT1 throughout many cell types and its abundance suggests its involvement in fundamental cellular function [10,13]. NEAT1 lncRNA has been associated with alternative pre-mRNA splicing in a number of cell types, but a role has not been linked to normal cellular events during differentiation. We hypothesized that NEAT1 levels would reflect the regulation of alternative splicing of two important splice variants for PPARγ1 and γ2, the main transcription factor driving differentiation of pre-adipocytes.

SRp40 (SFRS5) is a candidate protein for regulating PPARγ2 splicing. During differentiation, SRp40 levels are significantly increased on the day that splicing of PPARγ2 is induced. This SR protein was also shown to be relevant in insulin induction of PKCβII splicing [32]. There are several SRp40 binding sites within the PPARγ2 exon B. Figure 5 depicts the potential role of phosphorylated SR proteins in the splicing of PPARγ2. Exon B is not spliced into the PPARγ1 mRNA. Exon B’s inclusion in PPARγ2 results in an N-terminus different from PPARγ1. Exons A1 and A2 are not translated, and PPARγ1 translates as a shorter protein than PPARγ2. If an increased concentration of phosphorylated SRp40 protein is present after its release from NEAT1, this could signal the splicing of PPARγ2. Our data support this, as there is an increased concentration of phosphorylated SRp40 protein on Day 4.

Culturing pre-adipocytes in a cocktail of insulin, isobutyl methyl-xanthine, and dexamethasone induces adipogenesis in this model. In 3T3-L1 adipocytes, PPARγ1 is the first expressed transcription factor known to regulate differentiation, and the expression of PPARγ2 occurs a few days later [33]. The release of phosphorylated SRp40 during this crucial time in adipogenesis should result in nuclear reorganization with the splicing of PPARγ2. In Figure 5, we diagrammed the potential scheme of how NEAT1 tethering and association with SR proteins could result in titrating their nuclear concentration and phosphorylation for splicing prior to Day 4.

There are still many questions regarding paraspeckles and NEAT1 that remain a puzzle. The paraspeckle proteins now identified appear not to be involved in common cellular processes [20]. What is obvious, is that paraspeckles contain a wide variety of functionally non-related proteins (more than 30 nuclear proteins) involved in nuclear processes and separate them from other regions of the nucleus [20]. What is also known is that these proteins share classical RNA recognition motifs (RRMs) [20]. These motifs are common in SR proteins. Of interest here, is that paraspeckles disintegrate upon the depletion of NEAT1 transcripts [10,11,12,14]. Our observation that NEAT1 levels are at a nadir on day 4 of differentiation would support the notion that paraspeckles disintegrated during normal cell maturation. How this occurs, whether via transcription suppression or RNA decay, is yet to be investigated. Our data using siRNA to deplete NEAT1 also supports the notion that once NEAT1 is disintegrated, SR proteins are released to alter splice sites. We also see the transcription of NEAT1 increasing on Day 8, and SR proteins associating with the lncRNA. This would signal the restraint of certain SR proteins during maturation of adipocytes.

Mouse models lacking paraspeckles appear normal when raised under laboratory conditions and paraspeckles are not required for most differentiation processes [23]. Our results suggest a disorganization of paraspeckles which would accompany the nadir of NEAT1 on Day 4, to allow for differentiation of adipocytes to proceed. When adipocytes are fully differentiated, lncRNA levels of NEAT1 increase again. Observations in mouse models also point to the idea that there may be redundancy in lncRNA function with yet to be identified lncRNAs, or that paraspeckles form only briefly to regulate cellular events. It also suggests that lncRNA may have overlapping functions. MALAT1 is also thought to tether and titre SR proteins in cancer cells [34]. Other reports emphasize that regulation of lncRNA expression is linked to the modulation of specific nuclear bodies [35]. The experimental conditions used here will allow us to better understand the function of NEAT1 in controlling cellular function during development and metabolic processes.
